# Housing Horses in Individual Boxes Is a Challenge with Regard to Welfare

**DOI:** 10.3390/ani9090621

**Published:** 2019-08-28

**Authors:** Alice Ruet, Julie Lemarchand, Céline Parias, Núria Mach, Marie-Pierre Moisan, Aline Foury, Christine Briant, Léa Lansade

**Affiliations:** 1 INRA, UMR 85 Physiologie de la Reproduction et des Comportements, 37380 Nouzilly, CNRS, UMR 7247, 37380 Nouzilly, Université François Rabelais, 37041 Tours, IFCE, 49411 Saumur, France; 2 INRA, UMR 1313 Génétique Animale et Biologie Intégrative, 78352 Jouy-en-Josas, AgroParisTech, 75231 Paris Cedex 05, Université Paris-Saclay, 91190 Saint-Aubin, France; 3 INRA, UMR 1286 NutriNeurO, 33076 Bordeaux, Université Bordeaux, 33076 Bordeaux, France

**Keywords:** aggressive behaviour, animal welfare, horse welfare, housing system, individual boxes, stereotypies, stress, unresponsiveness

## Abstract

**Simple Summary:**

Horses are mainly housed in individual boxes. This housing system is reported to be highly detrimental with regard to welfare and could trigger the expression of four behavioural indicators of a compromised welfare state: stereotypies, aggressiveness toward humans, unresponsiveness to the environment, and stress-related behaviours. The aim of this study was to investigate whether several factors commonly observed in boxes (e.g., the presence of a window toward the external environment) and management practices (e.g., time spent being ridden) could alleviate the negative effects of individual boxes on welfare. The results show that the majority of the factors studied did not influence the expression of the indicators. In addition, the longer the horses spent in individual boxes, the more likely they were to express unresponsiveness to the environment. Overall, the main conclusion of this study is that the detrimental effects caused by the spatial, social, and dietary deprivations of this housing system could not be alleviated by small facilities in the box or changes in management practices. To preserve the welfare of horses, it seems necessary to allow free exercise, interactions with conspecifics, and fibre consumption as often as possible, to ensure the satisfaction of the species’ behavioural and physiological needs.

**Abstract:**

Horses are mainly housed in individual boxes. This housing system is reported to be highly detrimental with regard to welfare and could trigger the expression of four behavioural indicators of a compromised welfare state: stereotypies, aggressiveness toward humans, unresponsiveness to the environment, and stress-related behaviours. The aim of this study was to identify housing and management factors that could alleviate the detrimental effects of individual boxes on welfare. A total of 187 horses were observed over 50 days by scan sampling. The impact of 12 factors was investigated on the expression of the four behavioural indicators in three different analyses. The results show that the majority of factors tested did not influence the expression of the behavioural indicators. Only three (straw bedding, a window opening onto the external environment, and a reduced quantity of concentrated feed) would have beneficial, although limited, effects. Furthermore, the longer the horses spent in individual boxes, the more likely they were to express unresponsiveness to the environment. To preserve the welfare of horses, it seems necessary to allow free exercise, interactions with conspecifics, and fibre consumption as often as possible, to ensure the satisfaction of the species’ behavioural and physiological needs.

## 1. Introduction

The welfare of domesticated animals is increasingly important in our current society because of direct involvement in scientific, ethical, political, economic, and health issues [1,2]. Welfare can be defined as “the state of an individual as regards its attempts to cope with its environment” [3]. Coping refers to the successful adaptation of the animal to its environment, and the investigation of welfare involves assessing whether it is easy for the animal to cope and how large is the impact of the environmental conditions offered [4]. The concept of welfare thus refers to the animal’s perception of environmental challenges and depends on both the individual and the context: it is ascertained at a certain time for a specific subject living within a specific environment [5]. As defined by the five domains model [6], both environmental and animal-based measures are required to assess welfare [7]. Among the animal-based measures, behavioural indicators could determine the coping abilities of a subject and infer the affective experience resulting from the perception of the current situation [8,9,10]. In the literature on domesticated species, four main behavioural indicators have been identified as potentially reflecting a welfare deterioration: stereotypies, aggressiveness toward humans, unresponsiveness to the environment, and stress-related behaviours.

First, stereotypies and abnormal repetitive behaviours are probably the most common indicators shared by many species under sub-optimal captive conditions (sows: [11]; calves: [12]; lambs: [13]). These are repetitive and invariant behaviours with no obvious goal or function [14] that should always be considered as warning signs of potential suffering, even at a low frequency. Indeed, in stereotypies-eliciting situations, the low stereotypic animals could experience the worst welfare state [15]. In stereotypic horses, oral behaviours such as crib biting [16] or locomotion behaviours such as weaving [17] can be observed and potentially be resulting from frustration at not being able to satisfy natural needs such as social contact, free movement, and a continual intake of a fibrous diet [18]. Second, aggressiveness toward humans, as an indicator of a poor human–animal relationship [19] likely involving a compromised welfare state in farm animals (calves: [20]), can also be observed in equines [21]. In horses, aggressiveness is expressed by a variety of behaviours from ears pinned backward to biting or kicking and could emerge as a result of physical pain (injuries: [22]; chronic back pain: [21]). In addition to animal welfare concerns, sport horses are consistently handled for grooming, saddling, and bridling, which can be a relevant safety issues for human beings [23]. Third, unresponsiveness to environmental stimuli associated with a reduction in goal-directed behaviours would be signs of inadequate comfort and poor welfare, as is the case for cattle (calves: [12]; cows: [24]). In horses, a particular posture, called the “withdrawn posture”, has been described in horses experiencing unresponsiveness to the environment [25,26]: a similar height of the neck and back, with a fixed gaze and static ear and head positions [27]. This atypical posture is related to other symptoms characterizing depression disorders in human beings (e.g., expression of anhedonia: [28]). Finally, a wide range of stress-related behaviours can be observed depending on the species (cattle: [29]; sheep: [30]). In horses, an internal state of stress can be observed through an alert posture [31], indicating hypervigilance [32]. From a welfare point of view, the chronic expression of stress-related behaviours could compromise mental and physiological functions by generating inappropriate responses to environmental stimuli [33]. 

Evidence shows that the use of individual boxes as housing systems for sport horses could lead to the expression of the four aforementioned behavioural indicators, likely reflecting a welfare alteration. It has been proven that individual confinement could trigger the expression of both oral and locomotion-related stereotypies in many studies [34,35,36,37,38]. Being housed singly within individual boxes could also lead to the expression of aggressiveness toward humans in both young horses [39] and adults [34,40]. Horses individually stabled may also experience unresponsiveness to the environment expressed through the “withdrawn posture” [28,41]. Finally, total confinement in individual boxes has been associated with an increase in stress-related behaviours expressed through the alert posture [36,42].

Despite the apparent detrimental effects of the box environment on the welfare state, this remains the main type of housing system for sport horses in the field (32% to 90% of horses depending on the country; [43,44,45,46]), particularly for valuable ones. The main arguments in favour are related to practicality, safety, and comfort issues for caretakers and animals [47,48]. For these reasons, horses’ keepers are often reluctant to use another type of system, particularly group housing. However, the choice of certain facilities or management practices related to individual boxes may potentially alleviate the detrimental effects of this housing system on animal welfare. Highlighting these facilities or management practices could lead to recommendations that would allow owners to take effective action to preserve the welfare of horses while continuing to use this housing system. These factors can be grouped into four categories. The first category concerns the housing system features, such as the choice of bedding material. The second category includes feeding factors, such as the amount of hay and concentrated feed received per day. The third category concerns factors related to the type of equitation practices such as the riding discipline. Finally, the fourth category includes the intensity of the physical activity the horse participates in, such as the time spent being ridden per week. The impacts of some of these factors on horses’ welfare have already been studied in previous studies, but the results were sometimes controversial. For example, the link between a concentrated diet and the development of crib-biting has been widely highlighted [35,37,38], but the direction of the relationship has yet to be elucidated [18]. A non-straw bedding (e.g., wood shavings or pellets) increased both oral and locomotion-related stereotypies in some studies [38,49,50,51] but had no effect in others [34,52]. In addition, these studies generally focused on stereotypies as indicators of poor welfare but placed less emphasis on the three other behavioural indicators (aggressiveness toward humans, unresponsiveness to the environment, stress-related behaviours). However, a good welfare state is not necessarily guaranteed if a horse does not express stereotypies [15]. Indeed, he may also experience a compromised welfare state by expressing it in another way, such as recurrent aggressive behaviours, expression of the “withdrawn posture”, and stress-related behaviours [53]. These four indicators are thus essential to assess the welfare state as fully as possible. It is then possible that factors supposed to have no impact on the welfare state can actually have an influence. 

Moreover, most of the studies that were interested in the influence of a set of factors on the welfare state of horses have been mainly based on surveys administered to the horses’ handlers or short-term behavioural observations. It is then likely that the prevalence of behavioural indicators of a compromised welfare state were underestimated in these populations (e.g., survey results and assessment of stereotypies: [53,54]). However, others methods are usable in field conditions to assess some of the four aforementioned behavioural indicators, such as scan sampling [55] (stereotypies: [42,50,56,57]; unresponsiveness to the environment: [41]; stress-related behaviours: [42,58]). In a previous study [53], it was suggested that repeated observations regularly widespread over time, such as scans, could allow to maximize the chances of detecting stereotypic and aggressive horses, and thus limit underdetection. Scan sampling could also lead to the quantification of the expression of specific postures, such as the “withdrawn” and alert postures, allowing us to infer an overall internal state of animals over a given period of time. The apprehension of a persistent mental state is considered as particularly relevant with regard to the welfare assessment [5]. In addition, the scan sampling method has successfully permitted to reveal the impact of different kinds of environments on horses’ behaviour (e.g., influence of an enriched environment in horses living in individual boxes [59] or a progressive weaning method [60]). Finally, in view of the deleterious effects of individual boxes on the welfare state, it seems essential to identify the most sensitive individuals depending on inherent factors such as gender or age.

The aim of this study was to target specific individual (gender and age) and environmental factors related to housing and management practices associated with individual boxes that may influence the state of welfare. A total of 187 sport horses, restrictively stabled without access to paddocks or grazing turnouts, were studied. One trained observer used repeated scan sampling observations to assess the four aforementioned behavioural indicators. The observations were carried out for 50 non-consecutive days over a nine-month period.

## 2. Materials and Methods

### 2.1. Animals, Housing and Management Factors

The current study included 187 sport horses restrictively housed in individual boxes in four distinct barns within the same stable, without access to paddocks or pastures. All boxes measured ± 9 m^2^ and were cleaned six mornings out of seven. The individual characteristics as well as the housing and management factors were variable between horses. These variations are detailed below and were considered in the statistical analysis.

Individual factors. The individual characteristics of the horses are detailed in Table 1. The impact of gender (stallion, gelding, and mare) and category of age (mean ± SEM; 10.5 ± 0.25 years) were investigated. All individuals were Warmblood horses and came from related lines, so the breed influence could not be tested.

Housing factors. In the box, horses can have an open window allowing the observation of the external environment and other horses and/or a grilled window between two boxes allowing to benefit from visual and restricted tactile contact with the neighbouring animal. Moreover, different bedding material (non-straw or straw) can be used (Table 1). All these factors were tested in the statistical analysis.

Feeding factors. Horses received different quantities of concentrated rations, depending on their physical condition or activity. In addition, rations were distributed in three or four meals a day (Table 1). The impacts of these two feeding factors were investigated. Water was provided ad libitum by automatic drinkers, and all horses were fed with hay (9 kg in two meals per day), regardless of their needs. Thus, these factors could not be tested, even though an insufficient forage ration could lead to an increase in stereotypies [52], thereby affecting the expression of the behavioural indicators studied.

Equitation factors. Horses were trained in different disciplines (eventing, dressage, jumping) and for various levels of performance. They could engage in a low level of performance (amateur category), could be trained for high-level competitions (professional category), or have achieved the highest level of performance in international competitions or an equivalent level of training (expert category). These factors were investigated in the statistical analysis.

Factors of physical activity. Horses could participate or not in competition events and be ridden, trained on the lunge, or exercised with an automatic walker for varying durations per week. The impacts of these factors were studied. 

### 2.2. Assessment of the Behavioural Indicators Using Scan Sampling

A single experienced observer in equine ethology walked regularly in front of the individual boxes, at a distance of at least 1.5 m from the door making as little noise as possible. She observed the horse for 3 s and then recorded whether the animal expressed one of the behavioural indicators.

Each horse was observed for five scans per day on 50 non-consecutive days distributed over a nine-month period. Observations were equally distributed across times of the day (9:00 to 10:30, 10:30 to 12:00, 12:00 to 13:30, 13:30 to 15:00, and 15:00 to 16:30). The average number of total scans analysed per subject was 200 ± 18 (mean ± SD); variations in the number of scans resulted from the absence of the horse or the presence of the caretaker in the loose box at the time of the observation. Descriptive statistics regarding the four behavioural indicators are presented in Table 2.

### 2.3. Statistical Analysis

#### 2.3.1. Associating Behaviours with All the Individual and Environmental Factors

A non-metric multidimensional scaling (NMDS based on Bray-Curtis distances; metaMDS function, vegan R library) ordination method was used to visualize the dispersion and the relationships between the four behavioural indicators and all the factors studied. Due to the large number of null values, oral and locomotion-related stereotypies as well as aggressive behaviours were treated as binary data (Yes/No). “Withdrawn” and alert postures were retained as continuous variables, allowing the frequency of expression of these two indicators to be studied. A total of 20 iterations were carried out to produce the NMDS ordination matrix. The NMDS axis solution were selected as the best portrayal of the data when the final stress value was below 0.1. Subsequently, covariates of behavioural variation were identified by calculating the associations between the 12 factors studied and the behavioural ordination matrix with the envfit function in the vegan R library, with 10,000 permutations and Benjamin Hochberg multiple testing correction. The factors included were the gender, the age, the presence of an opening toward the external environment, the presence of a grilled window between boxes, the bedding material, the mean quantity of concentrated feed offered and the number of meal per day, the discipline along with the level of performance, the number of competing events performed during the study, and the time spent being ridden and being trained on the lunge/using an automatic walker. An adjusted *p*-value < 0.05 was considered as significant. This method enabled the selection of combined factors with the strongest correlation to behavioural variation. In addition, to quantify the fraction of the behavioural variance that could be inferred from the above-mentioned 12 factors, a constrained analysis was performed using an automatic stepwise model built with permutation tests (ordistep function, vegan R library). At each iteration, the covariate that contributed the greatest fraction of inferred variance was added to the factors added in the previous iterations. The function stopped when the model was not changed during one step. The statistical significance of the resulting estimate was evaluated using permutation testing with 10,000 permutations, in which for each permutation all 12 factors of each individual were assigned to a random individual, and then the entire analysis was rerun (including the feature selection procedure).

#### 2.3.2. Identifying Factors that Specifically Influence Each Behavioural Indicator

Another complementary approach was then carried out using linear mixed models to test the effects of the 12 factors on each behavioural indicator separately. Five distinct models were built with oral, locomotion-related stereotypies, aggressive behaviours, and “withdrawn” and alert postures as outcome variables. All models included the 12 aforementioned factors as fixed-effect parameters. Furthermore, horses were divided among four different barns within the same stable, so a barn effect was included as a random factor to encompass the variation among them. More precisely, the “withdrawn” and alert postures were analysed using linear-mixed effects models (LMMs; lmer function, lme4 R library) after applying a square root transformation to better approximate a normal distribution. These two models were fitted with restricted maximum likelihood (REML) and *p*-values (*p*) were derived using the Satterthwaite approximation (anova function, lmerTest R library), both produced acceptable Type 1 error rates [61]. Univariate analysis was first performed to assess the significance of each factor tested. Only variables showing an association with a significance of *p* ≤ 0.20 were retained in the final model. In addition, the age and the gender of the horses were systematically controlled for as potential confounding factors by quantifying the changes in coefficients and significances of other factors after removing and then reinserting them into the final model. A change of at least 10% in the value of at least one regression coefficient was considered to reflect a significant impact of the confounding factor, which was thus retained in the final model. Residuals were checked graphically for normal distribution and homoscedasticity. The proportion of variance explained by the fixed factors was also quantified using the marginal R^2^ (R^2^_GLMM(m)_%), as well as by both the fixed and random factors using the conditional R^2^ (R^2^_GLMM(c)_%) (r.squaredGLMM function, MuMln R library). Oral, locomotion-related stereotypies, and aggressive behaviours presented too positively skewed distribution due to a large number of null values. These behavioural indicators were thus analysed as binary data (Yes/No) using generalized linear mixed models (GLMMs) (glmer function, lme4 R library) fitted with binomial error distributions and the Laplace approximation for parameters estimation. Wald χ^2^ tests (wald.test function, aod R library) were carried out to test the fixed effects after checking for overdispersion [62]. The models were built in the same manner as LMMs. The goodness-of-fit of the final models was assessed using the Hosmer-Lemeshow χ^2^ test (hoslem_gof function, sjstats R library).

For all models, Tukey post-hoc tests (glht function, multcomp R library) were carried out to further investigate the effects of significant parameters.

All statistical analyses were performed using R software (version 3.6.0, R Development Core Team, Vienna, Austria, 2019) with a significance level of (*p*) ≤ 0.05.

### 2.4. Ethics Statement

The authors confirm that their study complies with the policy relating to animal ethic and the “3 Rs”. This study consisted in observing the horses under their current conditions of life. No specific interventions were done on the animals.

## 3. Results

### 3.1. NMDS and Constrained Ordination: Fitting of the 12 Factors onto the Behavioural Structure

First, the relationships between the overall behavioural structure and the 12 factors were investigated by conducting NMDS. A three-dimensional NMDS solution presented an accepted stress value of 0.08 (non-metric fit, R^2^ = 0.99; linear fit, R^2^ = 0.97). When fitting the 12 factors onto the ordination of the behavioural structure, age was significantly correlated with the NMDS ordination of the behavioural structure (envfit; R^2^ = 0.04, *p* = 0.01; Table 3). The remaining factors were not significantly correlated with the NMDS ordination of the behavioural structure. Complementarily, the automatic stepwise regression model built on the constrained ordination of behavioural indicators identified age (F(3) = 2.27, *p* = 0.02) and the presence in the box of an open window toward the external environment (F(3) = 2.13, *p* = 0.03; Table 4) as explanations for the behavioural profiles. The proportion of behavioural variance explained by all 12 factors was 8% (R^2^ = 0.08).

### 3.2. Mixed-Effects Models: Analysis of the Effects of the 12 Factors on Each Behavioural Indicator Separately

**Figure 1:**
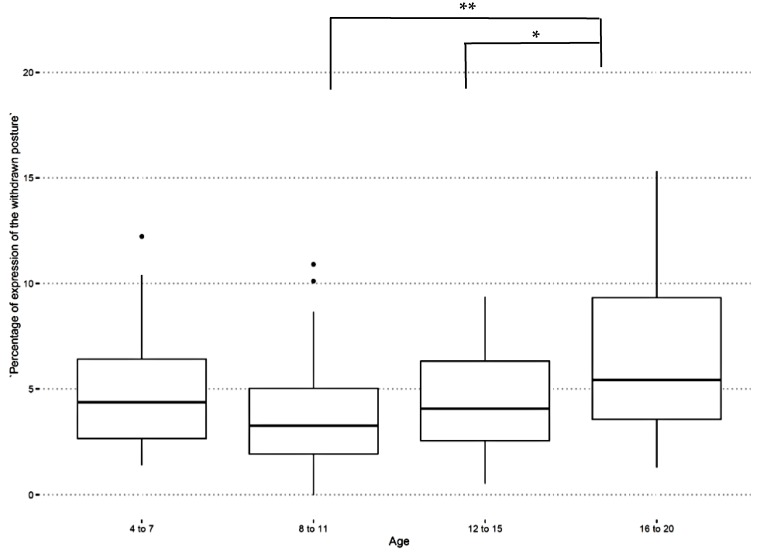
Percentage of expression of the “withdrawn posture” according to age categories. Horses aged between 8 to 11 and 12 to 15 were less unresponsive to the environment than those aged between 16 to 20 (Tukey post-hoc respectively: Z = 3.51, *p* = 0.002; Z = 2.62, *p* = 0.04). * ≤ 0.05; ** ≤ 0.01.

Individual factors. Among the individual factors, age had a significant effect on only the “withdrawn posture” (LMM: F(3) = 4.92, *p* = 0.003; Table 5). Post-hoc pairwise comparisons showed a significant increase in the frequency of this behavioural indicator with aging (Figure 1). Horses aged between 8 to 11 and 12 to 15 years showed less unresponsiveness to the environment than those aged between 16 to 20 years (Tukey post-hoc respectively: Z = 3.51, *p* = 0.002; Z = 2.62, *p* = 0.04; Table 5). Gender did not have a significant effect on the behavioural indicators (Table 5 and Table 6). 

Housing factors. Two housing-related factors have been shown to have a significant influence on aggressiveness toward humans (Table 6): the presence of a window opening toward the external environment (GLMM: χ^2^ (3) = 8.4, *p* = 0.038) and the type of bedding material (GLMM: χ^2^ (1) = 5.0, *p* = 0.026). Horses that had a window opening toward the external environment for the total duration of the study and kept in straw bedding were less aggressive compared to horses that never had this factor and were kept on non-straw bedding (Tukey post-hoc respectively: Z = −2.63, *p* = 0.04; Z = −2.23, *p* = 0.03). The type of bedding material also influenced the expression of alert postures (LMM: F (1) = 5.21, *p* = 0.027; Table 5). Horses kept on straw show a higher frequency of expression of this behavioural indicator compared to those kept on non-straw bedding (Z = 2.28, *p* = 0.02). The possibility for the horses to have reduced contact through a grilled window on the wall between two boxes did not have a significant effect on the behavioural indicators.

Feeding factors. The daily quantity of high-starch grain cereals received tended to trigger the expression of oral-related stereotypies (GLMM: χ^2^ (1) = 3.3, *p* = 0.067; Table 6). The number of meals per day did not have a significant effect on the behavioural indicators.

Equitation factors. None of the factors studied related to equitation (discipline and level of performance) had a significant effect on the behavioural indicators.

Factors of physical activity. None of the factors studied related to the regularity of the training (number of competing events the horses were in during the nine-month study period, time spent being ridden and trained on the lunge or walking on an automatic walker for weekly training) had a significant effect on the behavioural indicators. 

## 4. Discussion

Among the housing and management factors commonly observed in individual boxes, most of them did not significantly affect the welfare state of horses. Only three factors (straw bedding, a window opening toward the external environment, and reduced quantity of concentrated feed received daily) seem to be beneficial, but with limited effects. Above all, the longer horses live in individual boxes, the more likely they are to express persistent unresponsiveness to the environment.

### 4.1. Very Few Environmental Factors Influenced the Expression of the Behavioural Indicators

To alleviate the deprivations caused by individual boxes, it may be tempting to think that some factors regarding the housing system or the management practices would be beneficial for the welfare state of horses. This could have been the case, for example, by increasing physical activity through riding, lunging or using an automatic walker, or by providing specific facilities in the box, such as the presence of a grilled window allowing social contact. However, the results show that this would not be the case. Indeed, using three separate analysis, only three environmental factors seemed to significantly influence the welfare state of horses living in individual boxes, and these were limited to certain behavioural indicators, with a low proportion of explained variance.

First, the presence in the box of a window opening toward the external environment that allowed the horses to look outside and see other horses seemed to influence the expression of aggressiveness according to two separate analysis. This result is consistent with previous findings [63], suggesting that this factor could help to reduce frustration, which is sometimes expressed through aggressive behaviours toward humans [64]. The authors highlighted, however, that this box facility would not increase the overall level of satisfaction of the animals. Second, the choice of bedding material appeared to also influence the expression of aggressive behaviours. Indeed, horses kept on straw expressed less aggressiveness compared to those living on non-straw bedding. Several studies have already focused on the choice of bedding material, suggesting that straw bedding facilitates lying down, exploration, and food intake behaviours [50,65,66,67,68] compared to non-straw material. This could make it possible to better meet the behavioural and physiological needs of animals and reduce frustration and potential physical pain. Bedding material also appears relevant with regard to the welfare of other species (sheep: [69]; pig: [70]; cattle: [71]). It could thus be an interesting factor to take into account when using individual boxes as a housing system, particularly because aggressiveness can be a major safety issue for human beings [23]. The results of the study also showed an association between straw bedding and an increasing expression of alert postures, but this factor accounted for an explained variance in the model of only 4%, which is very low. Thus, it probably does not constitute a relevant influencing factor for this behaviour. Third, the quantity of the concentrated feed consumed per day tended to be related to the expression of oral-related stereotypies, supporting previous findings that found a positive correlation between concentrates consumption and crib-biting [35,37,72,73]. This relationship is supported by the fact that concentrated feeding stimulates the secretion of gastric acid in the stomach, which, in addition to low amounts of forage, could lead to gastrointestinal irritation and the development of oral-related stereotypies as an adaptive response [18]. Limiting the quantity of ingested concentrates could therefore prevent a welfare alteration. Overall, these three factors could be recommended to be taken into account when horses are strictly living in individual boxes.

However, no other factors were related to the expression of the behavioural indicators. This lack of effects is somewhat contradictory to the findings of previous works, which identified potential factors that could alleviate the detrimental impact of the box housing system on welfare, mainly on stereotypies. For example, housing factors such as straw bedding [49], a grilled window between two boxes [51,74] and an increase in concentrated rations from two to six meals per day [75] could lead to a decrease in the expression of several stereotypic behaviours. Similarly, the less horses are ridden (and in the western riding style or driven), the less likely they were to express certain stereotypies [34,49,72]. These discrepancies could be explained by the fact that, in these studies, the horses generally did not live strictly in individual boxes and had periods of free exercise in paddocks or pastures, sometimes with other horses. Thus, it is possible that the deterioration of the welfare state of those horses was less significant than in the current study. Indeed, the time spent in paddock had been linked to a decrease in stereotypies [51], aggressiveness [34,43], and stress-related behaviours [42]. It is thus suggested that, when horses are severely restricted in their movements, social contact, and, to a lesser extent, diet, as it is the case for those living strictly in individual boxes, these factors do not particularly limit the deterioration of the welfare state. 

The main relevant result of this study remains that the majority of the tested factors had no influence on the expression of the behavioural indicators, in particular on unresponsiveness to the environment and stress-related behaviours. This implies that drastic changes in the living and management conditions should be required to improve the welfare state of animals. Indeed, confinement in individual boxes imposes spatial and social deprivations that prevent animals from satisfying movement and social needs, for which they appear highly motivated (movement: [56,76]; social contact: [77]). On the contrary, other environments such as pasture with other horses could theoretically satisfy these species’ needs and thus restore an optimal welfare state. It was shown that horses living in long-term individual boxes experienced an improvement in their mental state through a positive cognitive bias when they were released in pasture with congeners [78], likely indicating a better overall welfare state. The absence of stereotypies in pasture was also already mentioned [79]. However, the relationship between stereotypies and welfare remains unclear, and they should not be used as the only behavioural indicators to assess the welfare state [15]. Thus, further studies are needed to assess the evolution of the four behavioural indicators to evaluate the impact on welfare as fully as possible after such changes in environmental conditions, and particularly for animals living in individual boxes over a long term. In practice, when it appears impossible to use pastures, at least providing an enriched environment with diversity in food, sensory stimulations and contact with conspecifics has been demonstrated to positively impact the welfare state of horses that are individually stabled [59,80].

### 4.2. The Longer the Horses Spent Time in Individual Boxes, the Greater the Risk That They Would Express a Persistent Unresponsiveness to the Environment

Beyond the effects of housing and management factors, a more general influence of age on the behavioural indicators was reported in the three analysis. In particular, an increasing expression of unresponsiveness to the environment was observed to be associated with increased age. A positive relationship between age and this behavioural indicator was already shown [25], and the authors suggested that this could be a sign of a particularly compromised welfare state. In the current study, the age is confounded with the time spent in individual boxes because the majority of horses were bought at three years old and lived in this condition from that age. It is thus likely that the observed unresponsiveness to the environment results from long-term spatial, social, and dietary deprivations caused by the individual boxes, indicating overall detrimental environmental conditions with regard to welfare.

One perspective raised by this study might be to approach the anxiety–depression continuum in horses, where stress-related behaviours potentially reflecting anxiety might be a prelude to a depressive-like state under persistent stressors [81]. Even though there were no statistically significant effects, the descriptive statistics presented in the Supplementary Materials (Table S1) show a decrease in the expression of the alert posture along with an increase in the expression of unresponsiveness over time. This would require a longitudinal study to be elucidated.

No gender effects were observed in this study. This result would mean that males (mostly geldings) and females would have a similar sensibility to being housed in individual boxes. An increased expression of abnormal behaviours in stallions has already been observed [52], but this difference was probably due to the isolation of these individuals from conspecifics, while the females remained in group. In the current study, stallions were housed and managed in the same way as geldings and females, which did not allow highlighting this effect.

### 4.3. Limitations and Perspectives

As welfare is a multidimensional concept [5], it appears then necessary to add physiological and health measurements to the assessment of the four behavioural indicators, to fully evaluate the effective long-term impact of the individual boxes on the welfare state of horses. Indeed, chronic stressors tend for example to decrease the immune responses [82]. It was suggested that serum cortisol, antibodies such as immunoglobulin A (IgA), and pro-inflammatory cytokine such as interleukin-6 (IL-6) could reflect serious psychological and physical stress in sows living on the long-term in confined conditions [83]. Identical measures could be implemented in horses. For example, lower baseline cortisol levels were observed in horses expressing the “withdrawn” posture, suggesting a disturbance of the hypothalamo-pituitary-adrenocortical axis [27]. In addition, more factors that potentially could alleviate the detrimental effects of the individual boxes on welfare could be tested in further studies, such as new feeding systems that are expected to improve adherence to the natural feeding behaviour of horses [84] or facilities allowing more social contact between animals [85].

## 5. Conclusions

This study showed that, for horses strictly living in individual boxes, only a few factors among the common facilities in the boxes and management practices influenced the expression of four behavioural indicators of a compromised welfare state (stereotypies, aggressiveness toward humans, unresponsiveness to the environment, and stress-related behaviours). These factors were the type of bedding material, the presence of a window opening toward the external environment, and the quantity of concentrated rations. These could be used to alleviate the detrimental effects of the housing system on welfare, with, however, limited effects. Overall, the main conclusion of this study is that these negative effects could not be significantly materially limited by small facilities in the box or changes in management practices. The implications of the results of this study for the welfare of horses are that it appears necessary to drastically change the living conditions of these animals, with the objective of promoting the satisfaction of the species’ needs. This kind of environment, such as pasture with other horses, could theoretically improve the welfare of horses. However, further studies are required to assess the evolution of the behavioural indicators and the welfare state of horses after the release is pasture, especially for animals living alone in individual boxes over a long-term. In addition, it appears essential to emphasize that strictly living in individual boxes remains detrimental for the welfare of horses, as it was also observed in this study that the longer the horses spent time in individual boxes, the greater the risk was that they would express a persistent unresponsiveness to the environment. The recurrent expression of this posture could reflect an internal state that is likely to be similar to depression in human beings.

## Figures and Tables

**Table 1:** Description of the 12 factors included in the analysis. The number of individuals (N) for categorical variables and the mean along with standard error (mean ± SEM) for continuous variables are presented.

**Categorical Variables**			
**Category**	**Factor**	**Mode**	**N**
Individual	Gender	Stallion	7
		Gelding	127
		Mare	53
	Age (years)	4 to 7	40
		8 to 11	73
		12 to 15	60
		16 to 20	14
Housing	Time spent in the box with a window opening on the external environment (out of the 9-month duration of the study)	0 month	48
		Between one week and 4 months	32
		Between 4 and 8 months	20
		9 months	87
	Presence in the box of a grilled window between two boxes	No	77
		Yes	110
	Bedding material	Non-straw	53
		Straw	134
Feeding	Meal of concentrated feed (number/day)	Three	101
		Four	86
Equitation	Discipline	Eventing	46
		Dressage	92
		Jumping	47
		Other (not analysed)	2
	Level of performance	Amateur	98
		Professional	34
		Expert	55
**Continuous Variables**			
**Category**	**Factor**		**Mean ± SEM**
Feeding	Quantity of the ration (kg/day)		3.52 ± 0.04
Physical activity	Number of competing events during the study		8.6 ± 0.92
	Riding (h/week)		5.7 ± 0.11
	Lunging and walking in an automatic walker (h/week)		1.9 ± 0.13

**Table 2:** Description of the behavioural indicators of the compromised welfare state in the sample. Stereotypies were divided into two sub-groups (oral and locomotion) because they could be influenced by different environmental factors. Descriptive statistics are presented as the percentage of horses expressing stereotypies and aggressive behaviours and the percentage of scans during which the “withdrawn” and alert postures were observed (mean ± SEM, Min–Max).

**Categorical Variables**			
**Behavioural Indicators**	**Sub-Group**	**Description**	**Percentage of Horses Expressing the Indicator**
Stereotypies	Oral	Crib-biting, Wind sucking, Clapping of lips, Tongue movements outside the mouth, Wood chewing	14.4%
	Locomotion	Head bobbing, Nodding, Weaving	18.7%
Aggressive behaviours		Simple threat (looking with ears pinned backward) Sustained threat (approaching with ears pinned backward and mouth open or turning hind quarters, sometimes raising a leg) Physical attack (bite or kick)	40.1%
**Continuous Variables**			
**Behavioural Indicator**		**Description**	**Percentage of Scans During which the Indicator was Observed**
“Withdrawn posture”		Neck horizontal at same level as back, fixed stare, ears and head static; [27]	Mean ± SEM: 4.5 ± 0.2% Min–Max: 0–24%
Alert posture		Elevated neck with ears pricked forward, looking intensely at the environment	Mean ± SEM: 1.3 ± 0.1% Min–Max: 0–13%

**Table 3:** The table displays the correlations between the 12 factors studied and the non-metric multidimensional scaling (NMDS) ordination of the behavioural structure via the envfit function. *p*-values are based on 10,000 permutations. For each factor, the table displays the direction cosines of the vector for each of the three axes, the R_adj_^2^ (squared correlation coefficient) of the relationship between the factors and the ordination and the permutation-based *p*-value. The three dimensions used in the NMDS resulted in a stress value of 0.08. ns: non-significant. Only age was significantly correlated with the behavioural structure, with a level accepted at *p* ≤ 0.05 (in bold).

Factors (Ordistep Function)	NMDS1	NMDS2	NMDS3	R_adj_^2^	*p* Model (>r)
Gender					
Stallion	−0.36	−0.22	−0.13	0.02	0.15 (ns)
Gelding	−0.05	0.05	0.002		
Mare	0.16	−0.09	0.01		
Age (years)					
4 to 7	−0.18	0.03	−0.02	**0.04**	**0.01**
8 to 11	0.01	−0.03	0.19		
12 to 15	0.16	0.03	−0.13		
16 to 20	−0.22	−0.04	−0.35		
Time spent in a box with a window opening on the external environment (out of the 9-month duration of the study)					
0 month	0.15	−0.13	0.03	0.02	0.17 (ns)
Between one week and 4 months	−0.007	0.04	0.10		
Between 4 and 8 months	0.06	−0.19	−0.08		
9 months	−0.09	0.10	−0.04		
Presence in the box of a grilled window between two boxes					
No	−0.12	0.02	0.008	0.009	0.17 (ns)
Yes	0.08	−0.02	−0.005		
Bedding material					
Non-straw	0.14	−0.04	−0.06	0.008	0.20 (ns)
Straw	−0.06	0.02	0.02		
Quantity of the ration	0.24	0.78	0.58	0.01	0.60 (ns)
Meals of concentrated feed					
Three	0.05	−0.01	0.003	0.002	0.72 (ns)
Four	−0.05	0.01	−0.003		
Discipline					
Eventing	0.13	0.05	0.07	0.009	0.54 (ns)
Dressage	−0.02	−0.001	0.006		
Jumping	−0.09	−0.04	−0.08		
Level of performance					
Amateur	−0.04	−0.02	−0.0004	0.005	0.77 (ns)
Professional	−0.03	−0.05	0.07		
Expert	0.09	0.06	−0.04		
Number of competing events during the study	0.52	0.65	−0.55	0.03	0.14 (ns)
Riding (h/week)	0.20	−0.77	−0.61	0.02	0.23 (ns)
Lunging and walking with an automatic walker (h/week)	−0.74	0.23	0.63	0.01	0.41 (ns)

**Table 4:** Automatic stepwise building on the constrained ordination of behavioural indicators with 10,000 permutation tests. The forward model selection was carried out on adjusted R^2^ and *p*-value with level accepted at *p* ≤ 0.05. The age and the presence in the box of an open window toward the environment had a significant effect on the total behavioural variance.

Factor	R^2^ Adjusted	Df	F Value	*p* (>F)
Age	0.02	3	2.27	0.02
Time spent in a box with a window	0.04	3	2.13	0.03
opening on the external environment				

**Table 5:** Fixed-effect parameters kept to build final models of “withdrawn” and alert postures after univariate analysis (linear-mixed effects models (LMMs) fitted with restricted maximum likelihood (REML) and Satterthwaite approximation). Marginal and conditional R^2^ quantify the proportion of variance explained by the fixed factors (R^2^_GLMM(m)%_) and both the fixed and random factors (R^2^_GLMM(c)_%). Gender and age were controlled for as confounding factors. The results of F-tests and Tukey post-hoc tests (Z values) are presented along with *p*-values (*p*). Values in bold represent significant parameters with level accepted at *p* ≤ 0.05. ns: non-significant.

Factor	Fixed-Effect Parameter	“Withdrawn Posture”	Alert Posture
	Marginal (R^2^_GLMM(m)_%) and conditional (R^2^_GLMM(c)_%) R^2^	R^2^_GLMM(m)_% = 0.12 R^2^_GLMM(c)_% = 0.15	R^2^_GLMM(m)_% = 0.04 R^2^_GLMM(c)_% = 0.24
Individual	Gender	ns	ns
	Age (years)	**F(3) = 4.92 (*p* = 0.003)**	ns
	“4 to 7” VS “8 to 11”	Z = −2.18 (*p* = 0.12)	
	“4 to 7” VS “12 to 15”	Z = −0.77 (*p* = 0.86)	
	“4 to 7” VS “16 to 20”	Z = 1.88 (*p* = 0.22)	
	“8 to 11” VS “12 to 15”	Z = 1.59 (*p* = 0.36)	
	“8 to 11” VS “16 to 20”	**Z = 3.51 (*p* = 0.002)**	
	“12 to 15” VS “16 to 20”	**Z = 2.62 (*p* = 0.04)**	
Housing	Time spent in a box with a window opening on the external environment (out of the 9-month duration of the study)	ns	ns
	Presence in the box of a grilled window between two boxes	F(1) = 2.84 (*p* = 0.09)	ns
	Bedding material	ns	**F(1) = 5.20 (*p* = 0.02)**
	“Non-straw” VS “Straw”		**Z = 2.28 (*p* = 0.02)**
Feeding	Quantity of the ration (kg/day)	ns	F(1) = 2.52 (*p* = 0.11), β = 0.01
	Meals of concentrated feed (number/day)	ns	ns
Equitation	Discipline	ns	ns
	Level of performance	ns	ns
Physical activity	Number of competing events during the study	F(1) = 1.13 (*p* = 0.29), β = 0.0005	ns
	Riding (h/week)	F(1) = 0.87 (*p* = 0.35), β = −0.003	ns
	Lunging and walking with an automatic walker (h/week)	ns	ns

**Table 6:** Fixed effect parameters kept to build final models of oral and locomotion-related stereotypies and aggressive behaviours after univariate analysis generalized linear mixed models (GLMMs) fitted with binomial error distribution and Laplace approximation). The goodness-of-fit of each model was assessed using the Hosmer-Lemeshow χ^2^ test. Gender and age were controlled for as confounding factors. The results of Wald χ^2^ tests and Tukey post hoc tests (Z values) are presented along with *p*-values (*p*). Regression coefficients (β) of the model are presented for continuous variables. Values in bold represent significant parameters with level accepted at *p* ≤ 0.05. ns: non-significant. _: No data available.

Factor	Fixed Effect Parameter	Oral-Related Stereotypies	Locomotion-Related Stereotypies	Aggressive Behaviours
Hosmer-Lemeshow		Χ^2^ (8) = 9.59 (*p* = 0.29)	_	χ^2^ (8) = 4.79 (*p* = 0.78)
Individual	Gender	ns	ns	χ^2^ (2) = 3.4 (*p* = 0.19)
	Age (years)	χ^2^ (3) = 6.5 (*p* = 0.09)	ns	χ^2^ (3) = 3.1 (*p* = 0.37)
Housing	Time spent in a box with a window opening on the external environment (out of the 9-month duration of the study)	ns	ns	**χ^2^ (3) = 8.4 (*p* = 0.038)**
	“0 month” VS “Between one week and 4 months”			Z = -2.07 (*p* = 0.16)
	“0 month”–“Between 4 and 8 months”			Z = -0.53 (*p* = 0.95)
	“0 month” VS “9 months”			**Z = −2.63 (*p* = 0.04)**
	“Between one week and 4 months” VS “Between 4 and 8 months”			Z = 1.25 (*p* = 0.59)
	“Between one week and 4 months”–“9 months”			Z = 0.054 (*p* = 0.99)
	“Between 4 and 8 months”–“9 months”			Z = −1.41 (*p* = 0.48)
	Presence in the box of a grilled window between two boxes	ns	ns	ns
	Bedding material	ns	ns	**χ^2^ (1) = 5.0 (*p* = 0.026)**
	“Non-straw” VS “Straw”			**Z = −2.23 (*p* = 0.03)**
Feeding	Quantity of the ration (kg/day)	χ^2^ (1) = 3.3 (*p* = 0.067), β = 0.90	ns	ns
	Meals of concentrated feed (number/day)	χ^2^ (1) = 0.25 (*p* = 0.62)	ns	χ^2^ (1) = 0.46 (*p* = 0.50)
Equitation	Discipline	ns	ns	ns
	Level of performance	ns	ns	ns
Physical activity	Number of competing events	χ^2^ (1) = 0.36 (*p* = 0.55), β = 0.01	ns	ns
	Riding (h/week)	ns	ns	χ^2^ (1) = 2.9 (*p* = 0.09), β = 0.21
	Lunging and walking with an automatic walker (h/week)	ns	ns	ns

## Supplementary Materials

The following are available online at https://www.mdpi.com/2076-2615/9/9/621/s1. Table S1: Descriptive statistics of the categorical factors in the analysis.
