# Supplementary material for: Housing Horses in Individual Boxes Is a Challenge with Regard to Welfare
Source: Animals (Basel). 2019 Aug 28;9(9):621. doi: 10.3390/ani9090621 (PMC6770668; doi:10.3390/ani9090621)
Supplement: Supplementary file 1 [file animals-09-00621-s001.zip › Supplementary materials.docx]

Table S1. Descriptive statistics of the categorical factors in the analysis

| **Factor** | **Mode** | **Oral-related stereotypies** | **Locomotion-related stereotypies** | **Aggressive behaviours** | **“Withdrawn posture”** | **Alert posture** |
| --- | --- | --- | --- | --- | --- | --- |
|  |  | Percentage of horses expressing the behavioural indicator | | | Percentage of scans during which the behavioural indicator was observed  Mean ± SEM  [Min – Max] | |
| Gender | *Stallion* | 14 % | 14 % | 28.6 % | 6.8 ± 2.9 %  [0.9 % – 24 %] | 2.7 ± 0.9 %  [0.4 % – 6.4 %] |
|  | *Gelding* | 16.5 % | 19.7 % | 36.2 % | 4.3 ± 0.2 %  [0 % – 15.3 %] | 1.2 ± 0.2 %  [0 % – 13 %] |
|  | *Mare* | 9.4 % | 17 % | 51 % | 4.5 ± 3.7 %  [0.9 % – 12.2 %] | 1.3 ± 0.2 %  [0 % – 5.2 %] |
| Age (year) | *4 to 7* | 17.5 % | 12.5 % | 30 % | 4.8 ± 0.4 %  [1.4 % – 12.2 %] | 1.5 ± 0.2 %  [0 % – 6.7 %] |
|  | *8 to 11* | 5.5 % | 27.4 % | 39.7 % | 3.7 ± 0.3 %  [0 % – 10.9] | 1.7 ± 0.3 %  [0 % – 13 %] |
|  | *12 to 15* | 21.7 % | 16.7 % | 50 % | 4.4 ± 0.3 %  [0.5 % – 9.3 %] | 0.9 ± 0.1 %  [0 % – 5.2 %] |
|  | *16 to 20* | 21.4 % | 0 % | 25 % | 7.6 ± 1.7 %  [1.3 % – 24 %] | 1.1 ± 0.4 %  [0 % – 5.5 %] |
| Time spent in the | *0 month* | 8.3 % | 18.7 % | 52 % | 4.3 ± 0.3 %  [0.9 % - 10.4 %] | 1.1 ± 0.2 %  [0 % - 8.7 %] |
| box with a window opening on the external environment | *Between one week and 4 months* | 12.5 % | 21.9 % | 34.4 % | 3.9 ± 0.7 %  [0.5 % - 24 %] | 0.9 ± 0.2 %  [0 % - 5.4 %] |
| (out of the 9-month duration | *Between 4 and 8 months* | 5 % | 5 % | 55 % | 4.2 ± 0.7 %  [0.5 % - 12.2%] | 1.1 ± 0.3 %  [0 % - 4.7 %] |
| of the study) | *9 months* | 20.7 % | 20.7 % | 32.2 % | 4.8 ± 0.3 %  [0 % - 15.3 %] | 1.5 ± 0.2 %  [0 % - 13 %] |
| Presence in the box of a grilled window | *No* | 11.7 % | 22.1 % | 35.1 % | 4.9 ± 0.3 %  [0.5 % - 15.3%] | 1.2 ± 0.2 %  [0 % - 10.7 %] |
|  | *Yes* | 16.4 % | 16.4 % | 43.6 % | 4.1 ± 0.3 %  [0 % - 24 %] | 1.3 ± 0.2 %  [0 % - 13 %] |

Table S1. Descriptive statistics of the categorical factors in the analysis (continued)

| **Factor** | **Mode** | **Oral-related stereotypies** | **Locomotion-related stereotypies** | **Aggressive behaviours** | **“Withdrawn posture”** | **Alert posture** |
| --- | --- | --- | --- | --- | --- | --- |
|  |  | Percentage of horses expressing the indicator | | | Percentage of scans during which the indicator was observed  Mean ± SEM  [Min – Max] | |
| Bedding material | *Non-straw* | 17 % | 22.7 % | 51 % | 4.7 ± 0.5 %  [0.5 % - 24 %] | 1.001 ± 0.2 %  [0% - 5.3 %] |
|  | *Straw* | 13.4 % | 17.2 % | 35.8 % | 4.4 ± 0.2 %  [0% - 12.2%] | 1.4 ± 0.2 %  [0% - 13 %] |
| Meal of concentrated feed (number / day) | *Three* | 10.9 % | 19.8 % | 44.5 % | 4.5 ± 0.3 %  [0.5 % - 15.3 %] | 0.9 ± 0.1 %  [0% - 6.5 %] |
|  | *Four* | 18.6 % | 17.4 % | 34.9 % | 4.5 ± 0.4 %  [0 % - 24 %] | 1.7 ± 0.2 %  [0% - 13 %] |
| Discipline | *Eventing* | 13 % | 26.1 % | 23.2 % | 4.7 ± 0.4 %  [0.9 % - 10.1 %] | 1.3 ± 0.2 %  [0 % - 6.5 %] |
|  | *Dressage* | 15.2 % | 17.4 % | 39.1 % | 4.3 ± 0.4 %  [0 % - 24 %] | 1.2 ± 0.2 %  [0 % - 13 %] |
|  | *Jumping* | 8.5 % | 12.7 % | 36.2 % | 4.6 ± 0.4 %  [1.06 % - 12.2 %] | 1.3 ± 0.2 %  [0 % - 4.9 %] |
| Level of performance | *Amateur* | 11.2 % | 18.4 % | 39.8 % | 4.4 ± 0.3 %  [0.5 % - 15.3 %] | 0.9 ± 0.1 %  [0 % - 5.4 %] |
|  | *Professional* | 14.7 % | 14.7 % | 38.2 % | 3.9 ± 0.5 %  [0 % - 10.4 %] | 1.7 ± 0.4 %  [0 % - 13 %] |
|  | *Expert* | 20 % | 21.8 % | 41.8 % | 5.003 ± 0.5 %  [0.5 % - 24 %] | 1.7 ± 0.3 %  [0 % - 10.7 %] |
